# Case Report: Three-dimensional characteristics of craniofacial morphology in facial asymmetry due to unilateral coronal synostosis

**DOI:** 10.3389/fdmed.2025.1622740

**Published:** 2025-10-29

**Authors:** Tomohiro Fukunaga, Shohei Shigemi, Masahiro Konno, Jun Uechi, Mitsuhiro Yoshizawa, Akiko Kishikawa, Yoshimichi Imai, Hideki Kitaura, Itaru Mizoguchi

**Affiliations:** ^1^ Division of Orthodontics and Dentofacial Orthopedics, Department of Community Social Dentistry, Tohoku University Graduate School of Dentistry, Sendai, Japan; ^2^ Division of Orthodontics and Dentofacial Orthopedics, Department of Oral Growth and Development, School of Dentistry, Health Sciences University of Hokkaido, Ishikari-Tobetsu, Japan; ^3^ Private Practice of Orthodontics, Hakodate, Hokkaido, Japan; ^4^ Private Practice of Orthodontics, Asahikawa, Hokkaido, Japan; ^5^ Department of Plastic and Reconstructive Surgery, Tohoku University Graduate School of Medicine, Sendai, Japan

**Keywords:** facial asymmetry, unilateral coronal synostosis, craniofacial morphology, three-dimensional (3D), glenoid fossa, mandible

## Abstract

This case report describes the three-dimensional (3D) craniofacial morphology of a patient with severe facial asymmetry caused by unilateral coronal synostosis. The patient was an 11-year-and-3-month-old girl at the time of the first examination. Facial photographs revealed upper facial deviation toward the right (affected) side and lower facial deviation toward the left (non-affected) side. The nasal bridge was bent toward the non-affected side, and the external canthus on the affected side was retracted superolaterally. The midline of the lower dentition deviated toward the non-affected side. Molar relationships were Class III on the affected side and Class I on the non-affected side. A virtual fusion model of the skull and dentition was reconstructed and analyzed using a 3D coordinate system. The model demonstrated absence of the right coronal and sphenofrontal sutures, deviation of the nasal pyramid and vomer toward the affected side, and anterior displacement of the petrous bone. Unlike typical facial symmetry cases, this case exhibited a prominently anterior glenoid fossa and reduced mandibular body length on the affected side. These findings demonstrate the complex craniofacial morphology associated with unilateral coronal synostosis and highlight the role of the coronal suture in maintaining facial symmetry and the mandible's adaptive growth in response to glenoid fossa asymmetry.

## Introduction

A recent meta-analysis reported a relatively high prevalence of facial asymmetry, ranging from 17.4% to 73.0% (1). As the severity of asymmetry increases, it negatively impacts both facial aesthetics and oral function (2, 3). Treating facial asymmetry presents a considerable challenge for orthodontists due to the involvement of complex skeletal imbalances and dental compensations (4–8). Although various etiological factors have been proposed including hereditary influences, deformational plagiocephaly, trauma, oral habits, functional mandibular shift, temporomandibular joint disorders, and abnormal condylar growth the exact causes of facial asymmetry remain poorly understood (9).

Craniofacial skeletal development involves predominantly sutural and intramembranous ossification, with endochondral ossification restricted to specific regions such as the cranial base, nasal septum, and mandibular condyle. Cranial sutures are synarthroses connecting the bones of the head with each other through a fibrous sutural ligament (10). Craniosynostosis is a condition in which the premature fusion of one or more cranial sutures leads to cranial deformation and associated clinical symptoms (11). Its prevalence is approximately one in every 2,000 live births (12). Craniosynostoses are primarily classified into two types: syndromic and non-syndromic. Syndromic craniosynostoses, such as Apert, Crouzon, Pfeiffer, and Muenke syndromes, constitute approximately 15% of all cases. These syndromes typically involve multiple sutural fusions and are characterized by well-established genetic causes (12). The remaining 85% of cases are classified as non-syndromic craniosynostoses, most of which involve the premature fusion of a single cranial suture. Although the precise etiology of non-syndromic craniosynostosis is unclear, several associated genes have been identified (12). Among non-syndromic cases, unilateral coronal synostosis is the most common, representing 12%–24% of cases (13).

Cranial deformation varies depending on the specific suture involved. For instance, unilateral coronal synostosis results in pronounced craniofacial asymmetry, including anterior plagiocephaly and flattening or recession of the forehead on the affected side (12). Although most craniosynostosis research has been conducted from medical perspectives, particularly in plastic surgery, neurosurgery, and pediatrics, limited information is available regarding craniofacial morphology and occlusion, which are essential for understanding the complexity of craniofacial asymmetry. A cephalometric study on unilateral coronal synostosis demonstrated that during growth, midfacial structures tend to rotate toward the affected side, whereas the lower face shifts toward the unaffected side, resulting in prominent facial asymmetry (14). Pelo et al. (15) investigated the three-dimensional (3D) craniofacial morphology of unilateral coronal synostosis cases using CT analysis; however, their study included patients who had previously undergone early fronto-orbital remodeling surgery, which could have influenced their craniofacial development.

Recent advances in 3D imaging technologies, including computed tomography (CT) and optical scanning, along with associated computer software, have enabled the acquisition of highly accurate and reproducible 3D data of the orofacial region in clinical settings (16–18). This case report presents the 3D craniofacial morphology of a patient with severe facial asymmetry resulting from unilateral coronal synostosis using a multimodal image-fusion technique. This method combined a virtual cranial model generated from CT imaging with a virtual dentition model obtained via optical scanning of dental casts (16).

## Case report

### Medical history and dental findings

The patient was an 11-year-and-3-month-old girl at the time of the initial examination. Her chief complaints were lower facial deviation and a dental midline shift. She had been delivered via vacuum extraction to healthy parents. Her medical history included asthma and strabismus. She had never exhibited cranial nerve symptoms, and craniosynostosis had not been suspected previously. At the age of 7 years, she was diagnosed with hemophagocytic syndrome and underwent steroid hormone therapy for 12 months. Genetic testing and evaluation for craniosynostosis were not performed, as the patient showed no syndromic abnormalities. Until her presentation at our clinic, she had never been diagnosed with unilateral coronal synostosis by either physicians or dentists.

**Figure 1:**
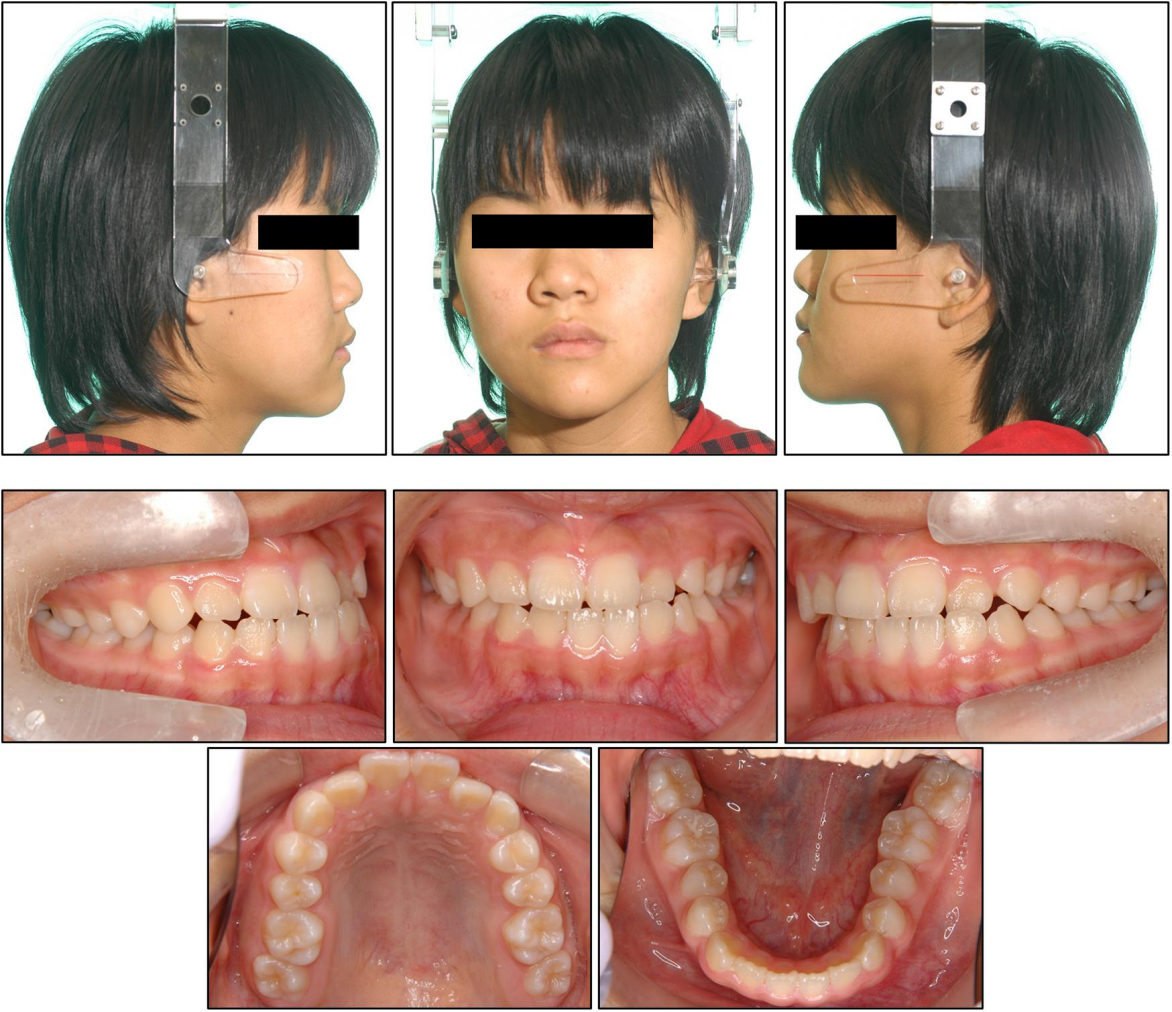
Facial and intraoral photographs obtained at the initial examination.

Facial photographs revealed that the upper face was twisted to the right, whereas the lower face deviated to the left (Figure 1). The nasal bridge bent to the left, and the external canthus on the affected side was retracted superolaterally. Her lateral profile appeared concave. The molar relationships were Class III on the right side and Class I on the left. Overjet and overbite measured 1.5 mm and 1.2 mm, respectively. The mandibular dental midline deviated 1.2 mm to the left relative to the maxillary midline. Arch length discrepancies were −1.2 mm in the maxillary dentition and −0.5 mm in the mandibular dentition. Crossbites were observed in the left lateral incisors and second molars.

**Figure 2:**
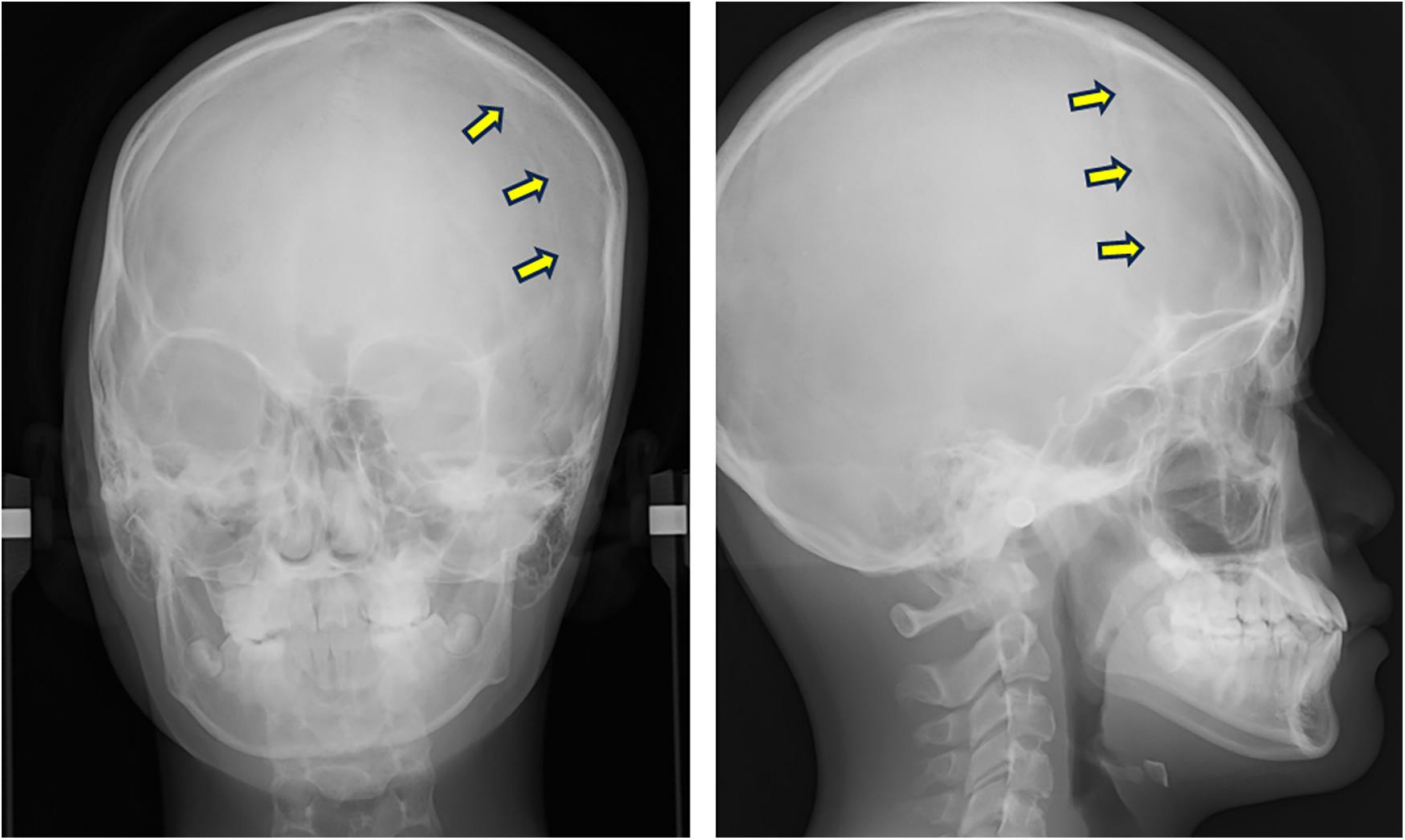
PA and lateral cephalograms taken at the initial examination. Arrows indicate the left coronal suture.

A posteroanterior (PA) cephalogram showed leftward deviation of the chin and a right downward cant of the occlusal plane (Figure 2). Lateral cephalometric analysis indicated a skeletal Class III relationship with a low mandibular plane angle (Supplementary Table 1). The maxillary incisors were labially inclined, whereas mandibular incisor inclination remained within the average range.

The patient was not concerned about upper facial asymmetry but expressed a strong desire to correct the deviation of the lower face and dentition. Given the severity of facial asymmetry and occlusal cant, a surgical orthodontic approach involving two-jaw surgery after completion of growth was planned. This treatment plan was accepted by both the patient and her parents.

### Methods for 3D analysis of cranial morphology

A virtual fusion model combining the skull and dentition was reconstructed and analyzed using a 3D coordinate system, as previously described (5, 16). The detailed reconstruction procedure is outlined below.

**Figure 3:**
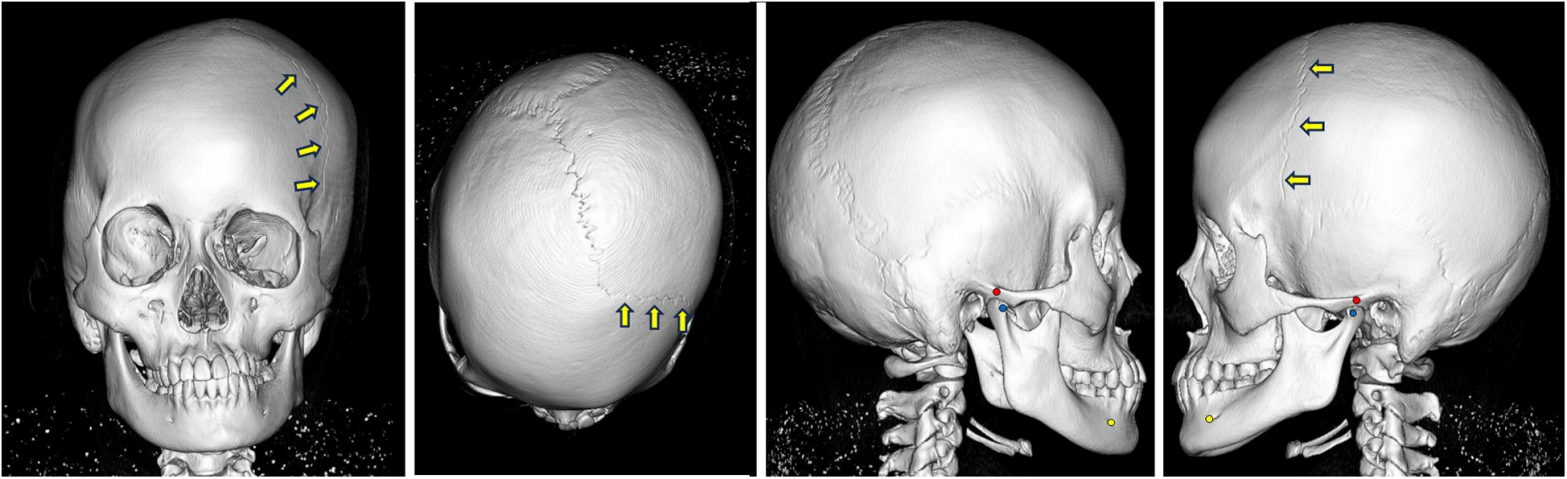
Virtual fusion model of skull and dentition at the initial examination. The coronal suture cannot be observed on the affected (right) side. Arrows indicate the coronal suture on the non-affected (left) side.

CT data of the skull were acquired using a helical CT scanner (Somatom Emotion 6; Siemens, Erlangen, Germany), and Digital Imaging and Communications in Medicine (DICOM) data were reconstructed using 3D imaging software (Dolphin 3D Image Software; GC Ortholy, Tokyo, Japan). Dental cast data were obtained using a 3D surface scanning system (Rexcan DS2; Solusnix, Seoul, Korea). The two datasets were then integrated by surface-based registration of the dentition (Figure 3). The resulting fusion model was segmented into four components: craniomaxillary complex, mandible, and upper and lower dentitions (5). A 3D coordinate system was established for each component to assess facial asymmetry. The midsagittal plane was automatically extracted using a surface-based method (19) (Supplementary Figure 1).

Initially, homogeneous surface areas were selected from either the left or right side of each segmented component—specifically, the periorbital bone for the craniomaxillary component, the bone around the mental foramen for the mandibular component, and the occlusal surfaces of the first molars for the upper and lower dentitions. These selected areas were horizontally mirrored and matched to the opposite side using an iterative closest point optimization algorithm. The midsagittal plane was then computed based on the positional relationship between the mirrored and original surface images. The horizontal planes were defined as perpendicular to the midsagittal plane and passed through the left Porion (Po) and left orbitale (Or) in the craniomaxillary component, the left mental foramen (MF) and left antegonial notch (AN) in the mandibular component, the incisal edge of the maxillary left central incisor (U1) and the mesiobuccal cusp of the maxillary left first molar (U6) in the upper dentition component, and the incisal edge of the mandibular left central incisor (L1) and the mesiobuccal cusp of the mandibular left first molar (L6) in the lower dentition component. The coronal planes were defined as perpendicular to the midsagittal plane, and passing through the left Po in the craniomaxillary component, the left mental foramen in the mandibular component, U6 in the upper dentition component, and L6 in the lower dentition component.

The 3D evaluation included: the relative attitude and position of the upper dentition to the craniomaxillary component; the relative attitude and position of the mandible to the craniomaxillary component; the relative position of the glenoid fossa to the craniomaxillary component; and dimensional analysis of the mandible in relation to the three coordinate planes. The relative attitude of each component was expressed as rotations along the orthogonal axes, specifically roll and yaw, while the relative position of the glenoid fossa was defined as the anteroposterior distance from the coronal plane (Supplementary Figure 2). Roll and yaw represent rotations along the anteroposterior axis and the vertical axis, respectively.

### 3D characteristics of craniofacial morphology

The virtual fusion model revealed the absence of the coronal and sphenofrontal sutures on the affected (right) side (Figure 3). Moderate lateral deviation of the nasal pyramid and vomer was evident, along with displacement of the petrous bone.

**Figure 4:**
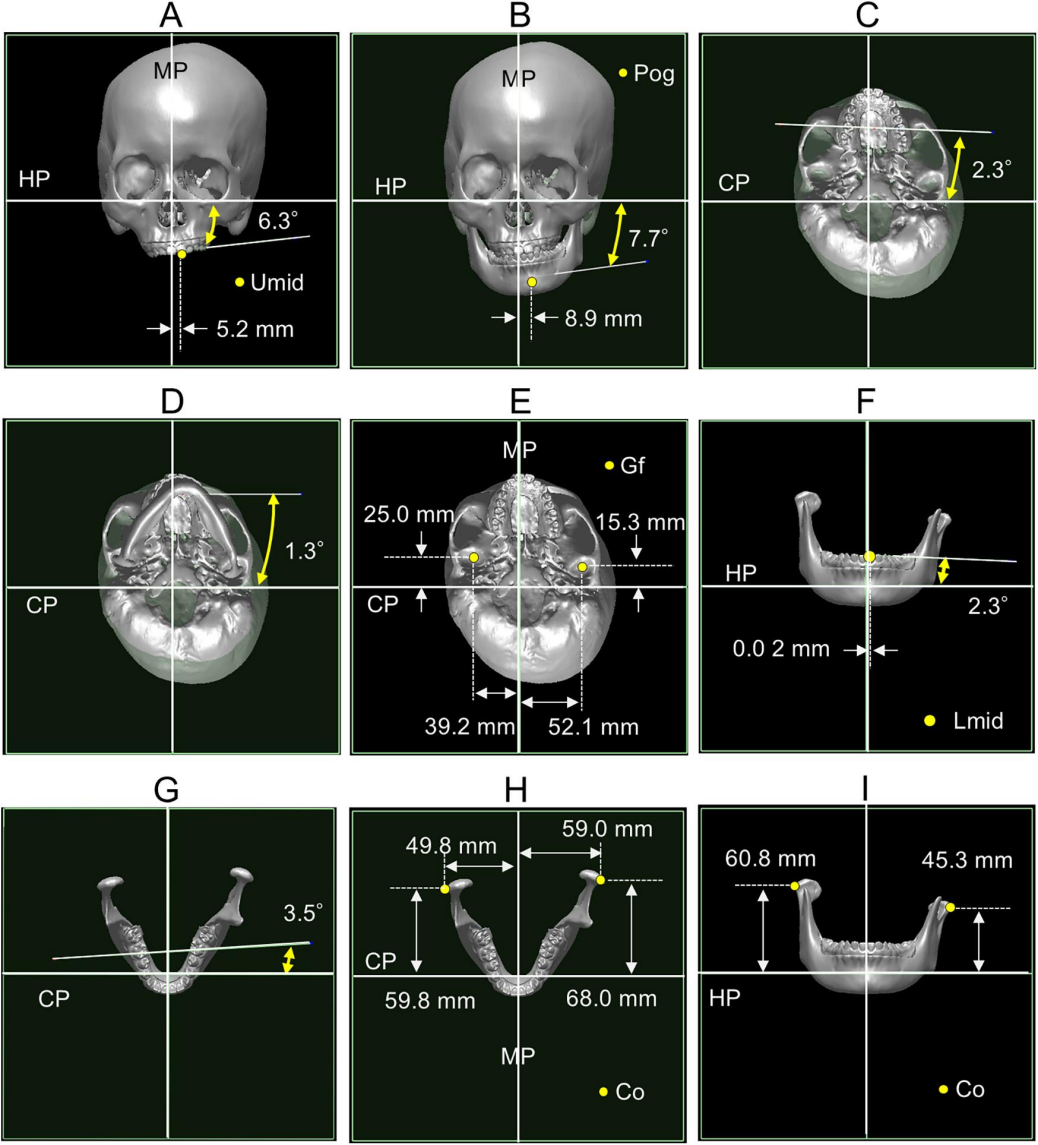
Three-dimensional cranial morphology. **(A)** Relative orientation and position of the upper dentition. **(B)** Relative orientation and position of the mandible. **(C)** Relative orientation of the upper dentition. **(D)** Relative orientation of the mandible. **(E)** Relative position of the glenoid fossa. **(F)** Relative orientation and position of the lower dentition. **(G)** Relative orientation of the lower dentition. **(H)** Mandibular length and width. **(I)** Ramus height. MP, midsagittal plane; HP, horizontal plane; CP, coronal plane; Umid, midline of the upper dentition; Pog, pogonion; Gf, glenoid fossa; Lmid, midline of the lower dentition; Co, outer pole of the condyle.

In the frontal view, the upper dentition exhibited a 6.3° counterclockwise roll and a 5.2 mm leftward deviation of the midline (Figure 4A). The mandible showed a 7.7° counterclockwise roll and deviated 8.9 mm to the left (Figure 4B). In the axial view, the upper dentition displayed a 2.3° counterclockwise yaw (Figure 4C); the mandible yawed 1.3° counterclockwise (Figure 4D). The glenoid fossa was located more anteriorly and medially on the affected side than on the unaffected side (Figure 4E). The lower dentition exhibited a 2.3° roll and a 3.5° yaw (Figures 4F,G). Dimensional analysis showed that mandibular length and width were smaller on the affected side than on the non-affected side (Figure 4H), whereas ramus height was greater on the affected side than on the non-affected side (Figure 4I).

## Discussion

In this case, syndromic craniosynostoses such as Apert, Crouzon, Pfeiffer, and Muenke syndromes were considered in the differential diagnosis. These syndromes are characterized by abnormalities of the head, neck, trachea, and limbs, although the location and severity of these abnormalities vary among syndromes and patients (12). Common features include multiple sutural fusions, hydrocephalus, proptosis, midface hypoplasia, upper airway obstruction, atlantoaxial dislocation, cervical vertebral fusion, and laryngotracheal malformations in the neck (20). Syndromic craniosynostoses were excluded in this patient, as she exhibited none of these features except midface hypoplasia. Based on CT imaging, the patient was diagnosed with non-syndromic unilateral synostosis.

This case presented with anterior plagiocephaly, a hallmark feature of unilateral cranial synostosis (11). The severity of anterior plagiocephaly is classified into four types based on radiological findings: type I, type IIA, type IIB, and type III (11). This case was categorized as type IIB, which is characterized by unilateral displacement of the petrous bone, as well as lateral deviation of the nasal pyramid and vomer. Previous reports have indicated that in coronal synostosis, not only the coronal suture but also adjacent sutures may be involved (21). Consistent with this involvement, the present case exhibited synostosis of both the right coronal and right sphenofrontal sutures.

We analyzed the craniofacial morphology of this case using a 3D virtual skull and dentition model in a patient with unilateral coronal synostosis and no history of fronto-orbital remodeling. The 3D model revealed several distinct morphological features, including canting of the maxillary occlusal plane and mandible, transverse deviation of the mandible, asymmetrical positioning of the glenoid fossa, i.e., a more anterior position on the affected (non-deviated) side compared with the non-affected (deviated) side, and dimensional discrepancies between the left and right mandibular components.

Among the features observed in this case, occlusal plane canting and mandibular deviation were consistent with findings from previous studies on facial asymmetry cases unrelated to unilateral coronal synostosis (5, 7). The asymmetry in glenoid fossa positioning has been reported in both unilateral coronal synostosis cases (15) and typical facial asymmetry cases without synostosis (6, 8, 22). Some researchers have noted a significantly anterior position of the glenoid fossa on the non-deviated side in facial asymmetry (6, 8, 22), whereas others have reported minimal or no relationship between glenoid fossa positioning and craniofacial asymmetry (7). These discrepancies may arise from differences in sample characteristics or 3D analysis methodologies. Facial asymmetry is often associated with complex and varied craniofacial disproportions; some cases exhibit mandibular dimensional discrepancies and others show asymmetrical glenoid fossa positioning (6, 8, 22).

Cranial sutures facilitate bone displacement in opposing directions by generating bone at the margins of adjacent bones, thereby accommodating the rapidly expanding brain. Anatomically, the coronal and sphenofrontal sutures contribute to anterior displacement of the frontal and adjacent maxillary bones and posterior displacement of the parietal and temporal bones. The glenoid fossa asymmetry observed in this case suggests that growth disturbances of the coronal suture and its adjacent sutures can influence the anteroposterior positioning of both the glenoid fossa and the mandible. Thus, disproportionate bone growth at the cranial sutures may play an etiological role in facial asymmetry.

Interestingly, the dimensional discrepancies between the left and right mandible contrasted with typical facial asymmetry cases, where the mandibular body is generally shorter on the deviated side (4, 7, 22). A longer hemimandibular body may serve to push the mandible toward the contralateral side. The condylar cartilage functions as a main growth site in the mandible, and exerts a dominant influence on craniofacial morphology and occlusion (23). The condylar cartilage is highly sensitive to changes in its environmental factors, such as biomechanical stress, hormones, and growth factors (23–25). Although the reason for this opposing trend in unilateral coronal synostosis remains unclear, the mandibular asymmetry observed may represent an adaptive response aimed at compensating for positional mandibular asymmetry (15).

Facial asymmetry often results from multifactorial disequilibrium involving both skeletal and dental components. It is conceivable that primary asymmetry in one structure induces compensatory adaptations in other structures to minimize the resulting imbalance, as demonstrated by the mandibular morphology in this case. Accordingly, a comprehensive understanding of 3D morphological characteristics is essential for accurate diagnosis and effective treatment planning in patients with facial asymmetry.

In cases of coronal synostosis with severe craniofacial deformities and life-threatening neurologic or respiratory symptoms, early surgical intervention followed by post-surgical monitoring is required (15). Currently, two-phase orthodontic treatment is recommended for patients with skeletal discrepancies. During the early mixed dentition stage, patients with mild to moderate asymmetries undergo the first phase of treatment using functional and fixed orthodontic appliances to correct mandibular skeletal and functional deviations, posterior crossbite, and dental midline shift (26). This is followed by a growth observation period. Careful monitoring is particularly important in patients with unilateral coronal synostosis, as they often show progressive worsening of facial asymmetry during pubertal growth (14). After puberty, the second phase of treatment—either orthodontic therapy alone or surgical orthodontic treatment—is selected depending on the severity of the skeletal asymmetry (2, 27).

A limitation of this report is that the observed skeletal characteristics are based on a single case. It remains unclear whether these features are consistently observed across different subtypes of facial asymmetry or how the maxillofacial skeleton responds to glenoid fossa asymmetry. Further clinical studies using 3D analysis in patients with facial asymmetry are warranted to clarify the relationship between glenoid fossa position and craniofacial morphology. Additionally, we plan to investigate glenoid fossa position and craniofacial morphology using an animal model with experimentally induced unilateral coronal synostosis (28).

## Conclusions

This case report provided a detailed 3D characterization of craniofacial morphology in a patient with unilateral coronal synostosis who had not undergone early surgical intervention. Analysis using a virtual skull and dentition model revealed anterior displacement of the glenoid fossa, reduced mandibular body length, and increased ramus height on the affected side. These findings highlighted the complex and asymmetric craniofacial architecture associated with unilateral coronal synostosis, demonstrating the value of 3D morphological assessment in understanding its anatomical features. Moreover, the 3D analysis system can be applied to surgical orthodontic treatment planning, particularly for simulating orthognathic surgery in patients with complicated and severe skeletal deformities such as this case. Although the asymmetrical position of the glenoid fossa cannot be corrected surgically, precise diagnosis and treatment planning based on 3D analysis may help achieve stable facial morphology and occlusion.

## Data Availability

The original contributions presented in the study are included in the article/Supplementary Material, further inquiries can be directed to the corresponding author.

## Supplementary material

Supplementary Figure 1 Midsagittal planes extracted using the surface-based method (upper row) and 3D coordinate systems (lower row). Blue and yellow patches represent reference images of anatomical structures used to determine the midsagittal plane. MP, midsagittal plane; HP, horizontal plane; CP, coronal plane.

Supplementary Figure 2 Terminology describing the relative attitude and position of cranial and dental components. Roll, rotation along the anteroposterior axis; yaw, rotation along the vertical axis; CP, coronal plane; Gf, glenoid fossa.
