# Supplementary figures and images for: Case Report: Three-dimensional characteristics of craniofacial morphology in facial asymmetry due to unilateral coronal synostosis
Source: Front Dent Med. 2025 Oct 29;6:1622740. doi: 10.3389/fdmed.2025.1622740 (PMC12605403; doi:10.3389/fdmed.2025.1622740)

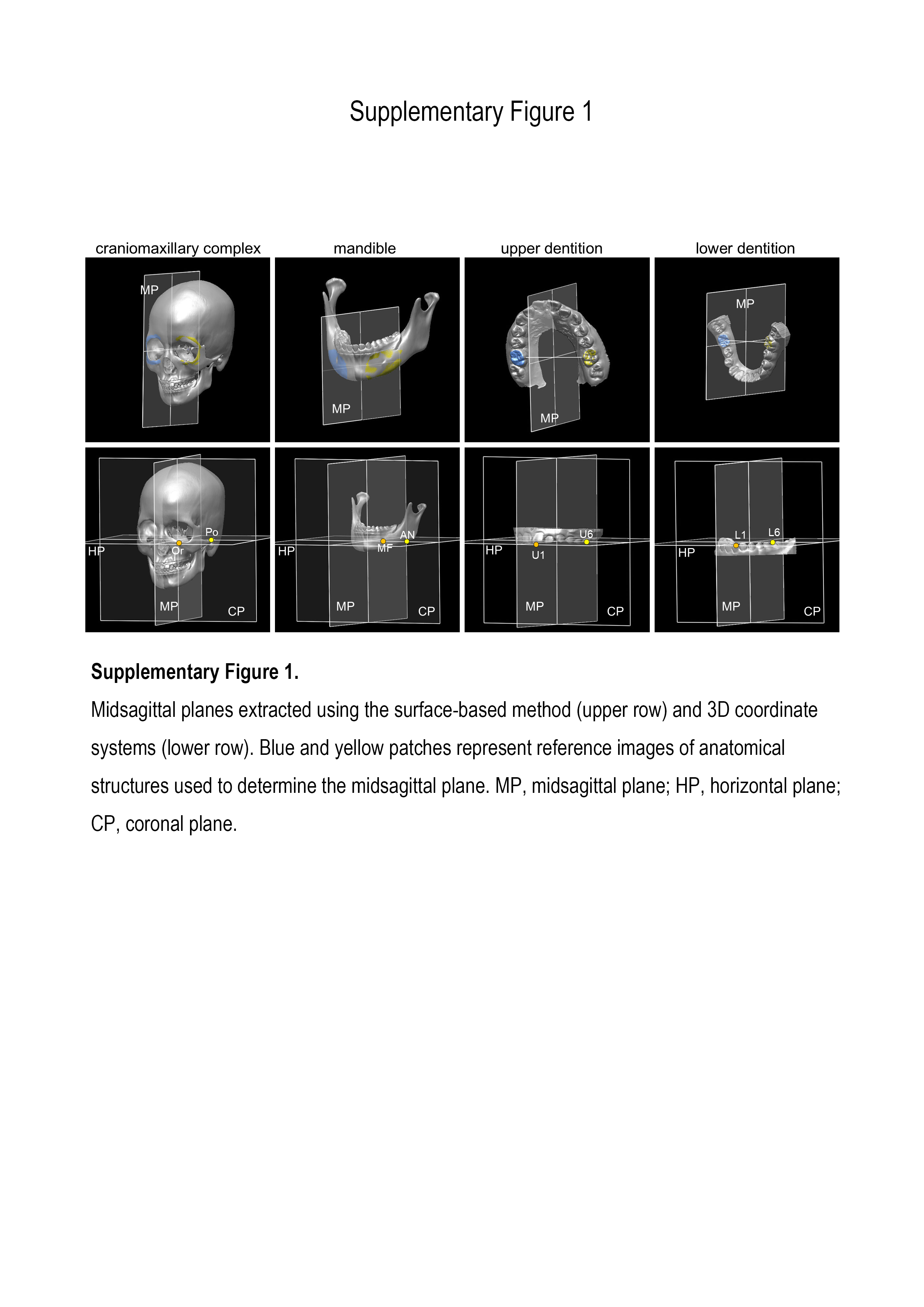

Supplement: Supplementary Figure 1 — Midsagittal planes extracted using the surface-based method (upper row) and 3D coordinate systems (lower row). Blue and yellow patches represent reference images of anatomical structures used to determine the midsagittal plane. MP, midsagittal plane; HP, horizontal plane; CP, coronal plane. [file Image1.tif]

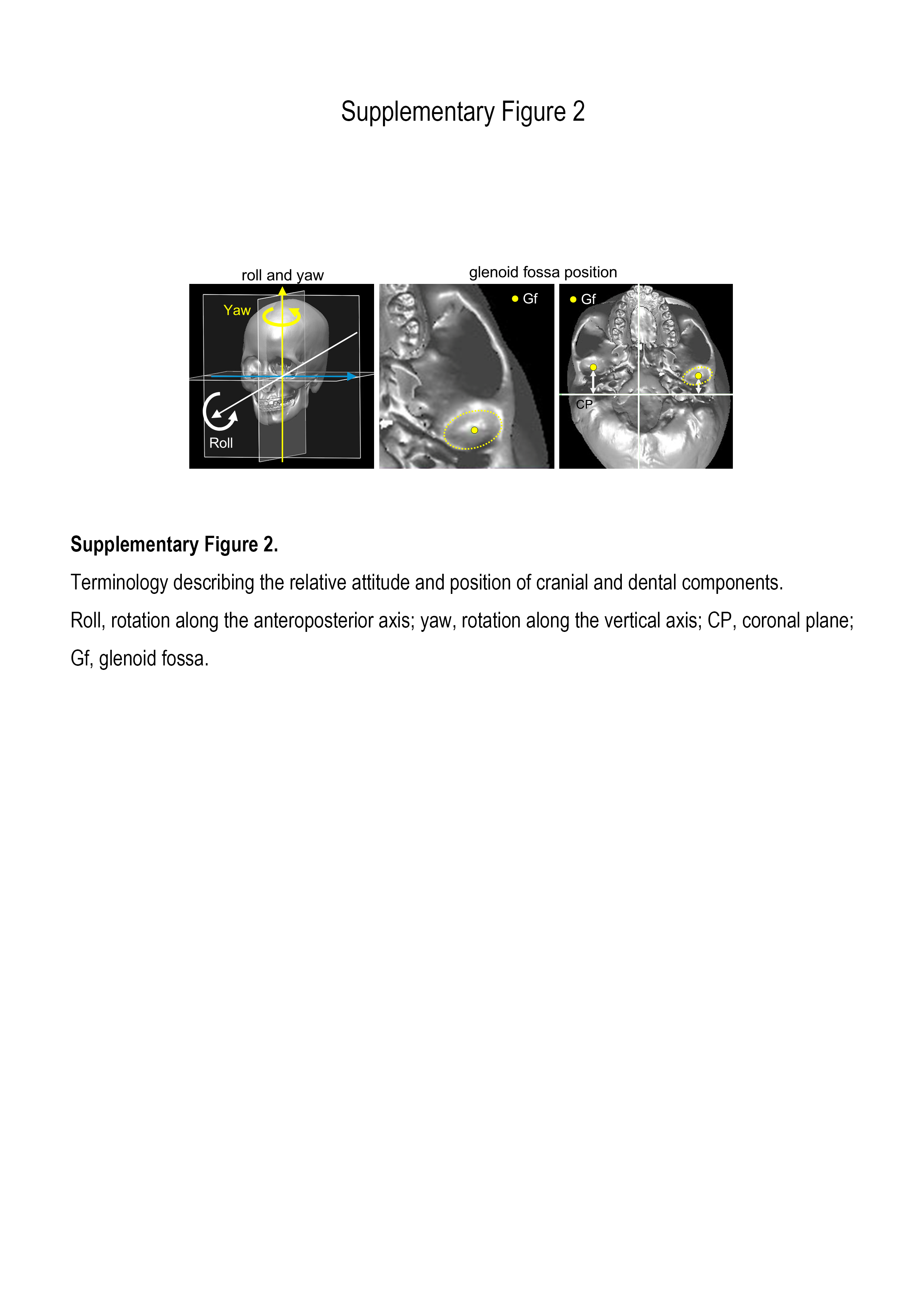

Supplement: Supplementary Figure 2 — Terminology describing the relative attitude and position of cranial and dental components. Roll, rotation along the anteroposterior axis; yaw, rotation along the vertical axis; CP, coronal plane; Gf, glenoid fossa. [file Image2.tif]
